# Nanostructured Hydrogels: A Method to Prevent Biofilms on Implantable Medical Devices

**DOI:** 10.3390/gels12020146

**Published:** 2026-02-05

**Authors:** Hasani G. Jayasinghe, Ujith S. K. Madduma-Bandarage, Sundar V. Madihally

**Affiliations:** 1 Math, Business, Science & Technology Division, University of New Mexico-Gallup, 705 Gurley Ave., Gallup, NM 87301, USA; hjayasinghe@unm.edu; 2 Department of Chemistry, New Mexico Institute of Mining and Technology, Lopez Hall 221, 801 Leroy Place, Socorro, NM 87801, USA; ujith.maddumabandarage@nmt.edu; 3 School of Chemical Engineering, Oklahoma State University, 420 Engineering North, Stillwater, OK 74078, USA

**Keywords:** hydrogels, biofilms, implants, bacteria, nanotechnology

## Abstract

Microbial biofilms pose significant health risks by causing infections associated with prosthetic and indwelling medical devices. Factors such as the high tolerance levels of biofilm microorganisms to antibiotics and the inability of antimicrobial agents to penetrate the biofilm matrix render antibiotic-based treatment methods ineffective against biofilm-related infections. Surfaces patterned with nanoscale topographical features have shown promising results in controlling the attachment of microorganisms. Therefore, nanopatterning of surfaces provides an excellent alternative to the existing antibiotic-based therapies. There are many techniques, such as photolithography and soft lithography, for patterning polymer or metal surfaces. However, depending on the cost, toxicity, feature size, and material compatibility, these methods have limitations. Although hydrogels have garnered special interest as biomaterials due to their biocompatibility and resemblance to the natural biological environment, hydrogels with surface nanopatterns have not been widely investigated as anti-biofouling materials. The applicability of hydrogels in biomedical applications and the importance of inhibiting microbial biofilms underscore the need for further research into the manufacturing of nanoengineered hydrogels with diverse topographical features. In this review, we discuss how nanostructured hydrogels inhibit biofilm formation. Further, we discuss nanopatterning methods, their limitations, advantages, and disadvantages. This article also highlights the current state of research on nanostructured hydrogels and associated challenges.

## 1. Introduction

Microorganisms such as bacteria and fungi naturally exist in two distinct states: planktonic cells or sessile aggregates (biofilms) [1]. In the planktonic state, microorganisms float freely. In the sessile state, they remain aggregated or attached to a surface, which can be either biological or non-biological. Biofilms can also be found on liquid surfaces, appearing as submerged or floating mats. A microbial biofilm can be defined as a coherent cluster of microbial cells embedded in a matrix and is more tolerant to most antimicrobial agents and host defense systems than the planktonic cells [1]. In sessile biofilms, these microorganisms attach irreversibly to the surface or other cells and secrete extracellular polymeric substances (EPS), including polysaccharides, lipids, proteins, and nucleic acids. The extracellular polymers facilitate the attachment of cells to one another and the formation of a matrix in which the cells remain embedded. In biofilms, multiple colonies of a single species or different species with varying functionalities can aggregate to form dense, organized communities [2]. The cells in the biofilm are genetically and physiologically different from their planktonic counterparts, as these organisms in biofilms exhibit slow growth rates, express genes involved in the production of EPS, and show high tolerance to antimicrobial agents and host defense systems [3,4].

Biofilms can grow on medical devices and implants that stay in the human body for prolonged periods, leading to device failure and/or severe health issues. These biofilm microorganisms are responsible for most chronic persistent infections, such as cystic fibrosis, periodontitis, rhinosinusitis, osteomyelitis, and kidney infections [2,4]. The low susceptibility of these biofilms to antimicrobial agents and the difficulties in penetrating antibiotics through the biofilm matrix render antibiotic-based therapies ineffective for treating biofilm-related diseases, resulting in severe health issues. Therefore, various other strategies have been investigated to prevent the formation of biofilms. Among these approaches, modifying the biomaterial surface with different topographical features has become an attractive option. As the global demand for prosthetics and medical devices is continuously increasing, device-related infections have become a serious issue in the healthcare and biomedical fields [4]. Thus, it creates additional research opportunities to develop biomaterials with improved antimicrobial/antiadhesive properties.

Hydrogels, a type of network polymer that can retain large amounts of water, have been extensively studied for their potential biomedical applications. Additionally, hydrogels are currently utilized in numerous applications as biomaterials. Thus, the formation of biofilms on the hydrogel surface creates problems and limits the use of hydrogels as biomaterials. Therefore, the fabrication of hydrogels that can prevent the formation of biofilms is essential. Nanopatterning has been explored for inhibiting biofilms on various materials, including metals, silicon, and polymers. However, studies investigating nanopatterned hydrogels as anti-biofouling materials are limited.

The antifouling properties of hydrogels have been discussed elsewhere [5]. A review by Takayuki Murosaki et al. detailed how marine sessile organisms avoid using seaweeds and fish as substrates for attachment, as these substrates contain hydrogels [6]. Also, other articles have been published focusing on the fabrication of antifouling surfaces for marine applications [7,8,9]. Here, they highlighted the chemical nature of the hydrogels that prevent attachment, such as the presence of hydroxy and sulfonic groups. Moreover, some articles focused on the antifouling activity of hydrogels against biofilms, particularly those based on nanomaterial-integrated coatings [10] and bioactive antifouling coatings. Another recently published article mentions the use of nanostructured hydrogels as biofouling materials, but it does not delve into nanostructured hydrogels [11]. Since other review articles discuss most other methods for modifying hydrogels to enhance their antifouling performance in detail, we intend this article to provide a comprehensive overview of nanostructured hydrogels, with particular focus on antifouling applications in biomaterials.

Rigid nanopatterned substrates can also facilitate the antifouling activity [12,13]. Although these rigid surfaces exhibit greater mechanical strength than hydrogels, their use in biological applications may be limited. Rigid substrates are more useful for marine or industrial applications [14,15]. Because hydrogels are more compatible with biological surfaces, this review focuses on strategies to enhance hydrogel properties to prevent biofilm formation, specifically through physical modifications. Here, we have discussed surface patterning techniques and their role in preventing microbial attachment. Also, the soft, water-rich structure of hydrogels physically blocks fouling organisms. Furthermore, it is easy to incorporate other modifications, such as nanoparticles to enhance antifouling activity, into hydrogels. However, their limited durability and reduced mechanical strength will limit the range of applications for hydrogels. There is also a current discussion of using hybrid methods to enhance the antifouling properties of materials [14].

## 2. Biofilms on Medical Devices and Implants

### 2.1. Formation of Microbial Biofilms

**Figure 1:**
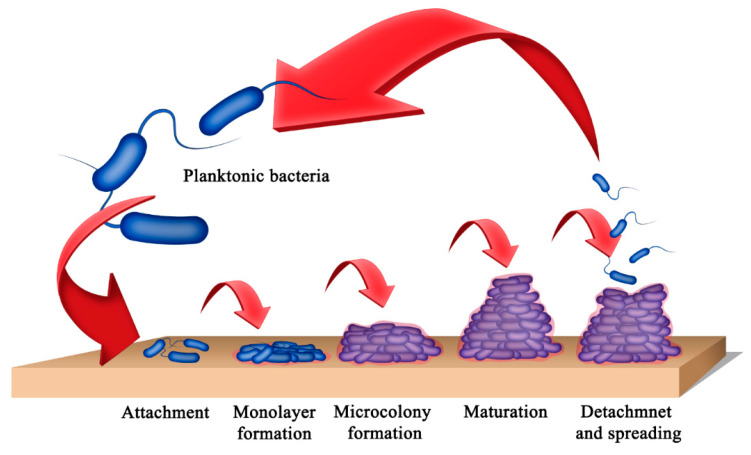
Stages in the formation of microbial biofilms on a surface.

A biofilm progresses through five different stages [3,16] (Figure 1): reversible/irreversible attachment of cells, formation of microbial colonies, formation of 3-dimensional biofilms, maturation, and detachment from the biofilm. Initially, planktonic microorganisms attach loosely to the surface. Initial attachment involves physical interactions such as electrostatic interactions, van der Waals forces, steric interactions, and appendages (pilli or flagella) of the microorganism [17]. Also, macromolecules, such as proteins, adsorb to the surface and form a conditioning layer, facilitating the attachment of microbes. The attached cells then secrete chemicals or extracellular polymeric substances (EPS) to strengthen their attachment to the surface (adhesion) and support attachment to one another (cohesion). The formation of EPS attracts cells and supports the formation of microbial colonies in the second stage. During the third stage, the cells proliferate rapidly to form 3-dimensional, multilayered colonies. These colonies further develop and subsequently reach the fourth stage. Biofilms can adopt different structures, such as flat plaques or mushrooms, depending on the availability of nutrients and other environmental factors [16,18]. The formation of multilayered biofilms also relies on the ability of the bacteria involved to attach to each other [17]. In the fifth stage, cells in the biofilm detach and return to the initial planktonic stage. The cells or cell clusters detach from the biofilm, either actively or passively, and disseminate onto a new surface, forming new colonies.

Attachment to surfaces and the formation of biofilms are advantageous for bacterial cells in many ways [19]. The channels in the EPS layer distribute nutrients and oxygen [20]. Some bacteria acquire metabolites and cofactors directly from the substrate [21,22]. The EPS provides mechanical stability and protection from mechanical damage and shear when attached to non-biological surfaces [23]. On biological surfaces, the extracellular polymeric matrix protects the cells in the biofilms from the host organism’s defense system and antimicrobial agents or antibiotics by acting as a barrier that prevents penetration of those attacking molecules to reach the embedded cells [16,24]. The depletion of nutrients and accumulation of waste in the biofilm cause changes in the microenvironment. Consequently, the cells in biofilms enter a slow-growing state and become less susceptible to the external factors that can attack them [2].

### 2.2. Biomaterials, Biofilms, and Influence on Human Health

Biofilms can grow on medical devices and implants that dwell in the human body for extended periods. These medical devices and implants include catheters, needles, pacemakers, endotracheal tubes, mechanical heart valves, prosthetic joints, contact lenses, and voice prostheses [16]. These biomaterials can become contaminated by bacteria and other microbes during the surgery or at any time during their stay in the body [4]. The most common types of microorganisms that form biofilms on medical devices are *Staphylococcus aureus*, *Staphylococcus epidermidis*, *Enterococcus faecalis*, *Escherichia coli*, *Streptococcus viridans*, *Klebsiella pneumoniae*, *Pseudomonas aeruginosa*, *Proteus mirabilis*, *Candida albicans*, and *Candida tropicalis* [16,25]. Table 1 summarizes different types of microorganisms that form biofilms on prosthetic and medical devices.

According to the estimates from the National Institute of Health, biofilms are responsible for more than 80% of microbial infections [42,43]. Biofilms can be formed by a single species (monospecies biofilms) or by a complex community consisting of organisms such as bacteria, fungi, and algae (multispecies biofilms) [44]. Table 2 provides a comparison of different biofilms.

Biofilm-associated infections can involve both Gram-negative and Gram-positive bacteria, e.g., oral cavity, diabetic foot infections, otitis media, and cystic fibrosis [56].

The biofilms grown on indwelling medical devices and implants pose detrimental health issues as they cause infections such as bloodstream infections and urinary tract infections, or device failure [16]. Moreover, cells detached from mature biofilms can spread to other organs, resulting in chronic infections [25]. The biofilm-related diseases include, but are not limited to, cystic fibrosis, otitis media, periodontitis, native valve endocarditis, chronic bacterial prostatitis, rhinosinusitis, osteomyelitis, non-healing chronic wounds, meningitis, kidney infections, and prosthesis and implantable device-related infections [4,17,57,58,59]. Additionally, these microorganisms are responsible for other medical conditions such as chronic inflammation and delayed wound healing, etc. [18,60]. Generally, antibiotics have low efficacy against biofilm-related infections due to decreased susceptibility of the microbes in biofilms to antibiotics and the failure of antimicrobial agents to penetrate the biofilm matrix [61]. Also, antibiotics and their doses selected based on the phenotype of planktonic cells of the pathogen may not effectively treat the biofilm-related diseases due to the different phenotypes of cells that grow in biofilms [2]. The treatment methods for these infections mostly require prolonged antibiotic therapy and/or surgical procedures to replace the infected device. Also, the microorganisms can re-colonize and form biofilms on the new implant or device [62]. The complexity and ineffectiveness of treating biofilm-related infections necessitate exploring alternative strategies to prevent biofilm formation on medical devices and implants.

## 3. Strategies That Can Prevent the Formation of Biofilms

**Figure 2:**
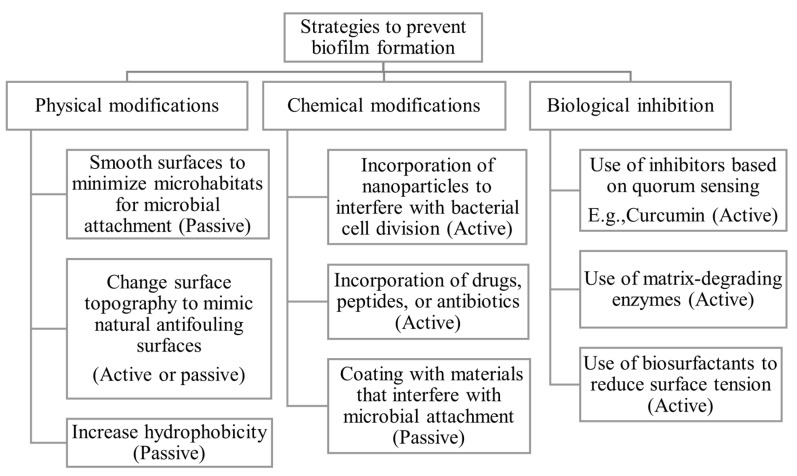
The classification of strategies used to combat biofilms on biomaterials.

In general, the methods used to combat biofilms involve different strategies: prevention of adhesion of microbes, disruption of the extracellular matrix of premature biofilms, killing the cells, and degradation of mature biofilms [63,64,65]. The methods that inhibit biofilms on biomaterials, as shown in Figure 2, can be classified as physical, chemical, or biological methods [66,67,68]. These approaches inhibit the formation of biofilms passively or actively [69]. The passive strategies target the prevention of adhesion of microbial cells onto the biomaterial surface without interfering with the biological activities of related microorganisms, whereas the active methods kill the microorganisms [69].

A convenient way to eradicate biofilms on medical devices and prostheses is to prevent the initial attachment of microorganisms onto the biomaterial surface. Attachment of microorganisms onto a biomaterial surface can be interfered with by tailoring the surface properties, such as surface energy, topography, functional groups, hydrophobicity/hydrophilicity, roughness, stiffness, and charge [70,71,72].

The most common surface modification techniques that inhibit biofilms on medical implants include anti-biofouling coatings generated using biomolecules, zwitterionic polymers, hydrophobic polymers, and hydrophilic polymers, or surface grafting [25,71,73,74,75,76,77,78]. Several authors have reviewed these approaches [4,25,70,79], and therefore, are not included in this review.

Various methods used to combat biofilms on biomaterials have advantages and disadvantages. Table 3 summarizes the advantages and disadvantages of these methods [67,80,81,82].

## 4. Nanotechnology-Based Methods to Prevent the Formation of Biofilms

As an alternative to conventional techniques, nanotechnological methods have been extensively studied for preventing biofilms on biomaterials. The nanotechnology-based approaches mainly include the incorporation of nanoparticles into a biomaterial or the modification of the biomaterial surface with nanoscale topographical features [83].

### 4.1. Incorporation of Nanoparticles

The use of nanoparticles to combat biofilms has been extensively studied and reviewed recently [3,84,85]. Different types of nanoparticles include metals, metal oxides, lipids, peptides, and polymers or composites of these types [3,86]. Furthermore, these nanoparticles can be categorized as antiadhesive nanoparticles, biocidal nanoparticles, bioactive nanoparticles, reactive oxygen species-releasing nanoparticles, and stimuli-responsive nanoparticles based on their biofilm prevention mechanism [3]. Application of nanoparticle-based therapies to control biofilms has several advantages: successful penetration into the biofilms, preferential delivery of drugs/antibiotics to the target site, controlled delivery of antimicrobial agents for extended periods, delivery of multiple agents for combined treatment, and improved efficacy [58]. Although nanoparticle-based approaches show promising results in controlling microbial biofilms, they also have some drawbacks. For example, the emergence of microbial strains resistant to antimicrobial agents such as silver and copper is the main disadvantage [87,88]. Also, the antimicrobial agents in the storage can be depleted over time, decreasing the effectiveness of the treatment [89].

Mechanisms of biofilm inhibition by nanoparticles include the generation of reactive oxygen species (ROS), the inhibition of quorum sensing, the degradation of the biofilm matrix, and enhanced drug permeability [90]. Nanoparticles accumulated on the bacterial cell membrane increase the bacterial permeability. Moreover, metallic nanoparticles can generate ROS that can cause oxidative damage to cellular components, such as DNA, lipids, and proteins. Metallic ions released from nanoparticles can disrupt DNA replication by reducing intracellular ATP [91].

Nanoparticles also inhibit Quorum Sensing, the molecular communication system essential for colony growth and biofilm development [92]. Table 4 summarizes the mechanisms by which nanoparticles inhibit biofilms.

### 4.2. Surface Patterning, Nanotopography, and Microbial Attachment

Although the chemical modifications improve the antimicrobial properties of the biomaterial, they may affect the biocompatibility and other favorable properties [106,107]. In contrast, patterning surfaces with different topographical features is a physical modification that does not affect the chemical, physical, or biological properties of the bulk material [108]. However, surface patterns determine the surface properties of the material that control interactions between microorganisms and the material. For instance, the introduction of nanotopographical features alters the surface stiffness, which is crucial for interacting with biological entities, without affecting the bulk chemical properties of the substrate. Therefore, in biomaterials, surface patterning is used to facilitate the integration of host tissues at the implantation site and the synthetic biomaterial for optimum functionality [109].

Since the surface texture determines the surface properties, the attachment of microorganisms on materials with different surface topographical features has been extensively studied [110,111,112,113]. The studies are based on three main categories of surface topographies: irregular or random patterns, regular or defined patterns, and hierarchical structures [110]. Random topographies lack properly defined dimensions. In contrast, regular patterns have clearly defined topographical features. Hierarchical structures contain two or more types of topographical features with varying dimensions.

Numerous studies investigate the attachment of microbes to surfaces lacking well-defined surface features. The surfaces are subjected to various treatments, including chemical etching and mechanical roughening, to alter their properties, including surface energy and roughness. Surface roughness was used as the main parameter to describe the differences between the surfaces [114,115]. In one study [116], nanorough titanium substrates, prepared by the electron beam evaporation technique, exhibit reduced attachment of *S. aureus*, *S. epidermidis*, and *P. aeruginosa* compared to flat controls. The same study revealed that the method used to create nanostructures also plays a vital role in controlling bacterial attachment. Here, the nanotubular and nanotextured titanium substrates generated by the anodization processes increased the bacterial adhesion. Another study [117] reports a correlation between the roughness and the attachment of *S. aureus* and *E. coli* onto titania surfaces. In this study, the patterned surface was generated by the supersonic cluster beam deposition method. As the roughness increased from ~16 nm to ~32 nm, the attachment of bacteria and the formation of biofilm were decreased. In another example [118], the titanium surfaces treated to obtain ultra-fine grain sizes promoted the adhesion of *S. aureus* and *P. aeruginosa.* Additionally, nanophase titania surfaces with high roughness in the nanometer regime demonstrate the increased attachment of *Pseudomonas fluorescens* and *Pseudomonas putida* [119]. Moreover, various studies report that a clear relationship does not exist between bacterial adhesion and the surface roughness of different materials such as glass, stainless steel, and metal oxides [120,121,122,123]. Contradictory results and a poor understanding of how these random topographies affect microbial attachment can be attributed to limitations in evaluating dimensions of random structures, variation in roughness measurements, and physiological differences among the tested bacteria [20,110].

**Figure 3:**
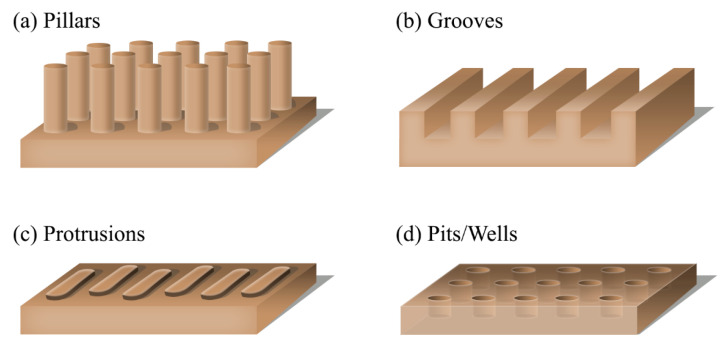
Types of regular nanotopographies.

As explained above, surface roughness does not correlate with microbial attachment. Therefore, it is crucial to investigate the attachment of microorganisms onto surfaces with defined structures. Several studies have investigated the attachment of microbes to micro/ nanoengineered surfaces with various topographies, including wells or pits, [112,124] pillars, [89,106,125] protrusions, [108,126] and grooves, [127,128] (Figure 3). These surface topography features at micron and submicron levels show profound effects on the attachment of microorganisms such as bacteria and fungi [126].

Unlike on a flat substrate, the entire surface is not accessible to the cells on a patterned surface. Here, the cells can access only the feature tops; hence, the available surface area for bacterial attachment decreases. The adhesion of Gram-positive, round-shaped *S. aureus* (~1 μm) and *S. epidermidis* (~1.5 μm) was significantly decreased when the surface of polyurethane substrates was patterned with nanopillars of diameters ~400–500 nm and heights ~600 nm–700 nm [106]. It is noteworthy that the feature sizes are smaller than the cells; thus, the cells experience reduced surface area, resulting in lower attachment. Similarly, the silica substrates containing circular (diameter 500 nm) or rectangular (1 × 1.5 μm or 1 × 2 μm) wells resulted in low attachment of Gram-negative *E. coli* (pathogenic and nonpathogenic strains) and *P. fluorescens* compared to the unpatterned controls [112]. In contrast, Gram-positive *Listeria innocua* showed attachment patterns similar to those on patterned silica surfaces and on the smooth controls. On the other hand, alumina (ceramic) membranes containing 20 nm and 200 nm pores showed lower attachment of nonpathogenic *E. coli* strains and *L. innocua* than the smooth control samples. Pathogenic *E. coli* and *P. fluorescens* showed an opposite trend with low attachment on the smooth alumina substrates [112]. The opposing trends confirm that attachment depends on the type of bacteria, the material, and the surface topography. According to the same study, the surface topography influences the morphology of bacterial cells, and the cells exhibited a preferential orientation to maximize the interactions with the substrate [112].

When the surface textures and gaps are smaller than the microbial cells, the entire cells cannot lie on the features or fit into the gap. Instead, the cells align according to the pattern [129] or stay across the trenches connecting neighboring features as a bridge [127]. Therefore, microbes can be preferentially aligned by adjusting the feature size and the gap. For instance, arrays of high-aspect-ratio (HAR) nanoposts generated on epoxy substrates demonstrated a guided alignment of *P. aeruginosa* cells [89,129]. The bacterial cells were oriented along the posts, normal to the surface, when the spacing between adjacent posts (~0.9 μm) was smaller than the rod-like *P. aeruginosa* cell (~1.2–1.5 μm). The cells lay on the surface when the spacing was close to the length of a cell. Similarly, *E. coli* cells preferentially settled down in the spacings or valleys between the square protrusions on poly(dimethyl siloxane) (PDMS) substrates [130]. The cells are positioned in the valleys when the valleys are larger than the protruding feature. The cells stayed on top of the large protrusions (feature size > 20 μm × 20 μm) because the protrusions are large enough to hold the cells [130]. A similar study [124] reports the evaluation of the retention of *P. aeruginosa*, *S. aureus*, and *C. albicans* on silicon wafers coated with titanium substrates. The substrates contained pits of varying diameters (ranging from 0.2 μm to 2 μm). *S. aureus* (1 μm) showed the highest retention, whereas *C. albicans* (4 to 5 μm) had the lowest retention. It is clear that the retention of microbes depends on the size of the pit and the size of the cell [130].

**Figure 4:**
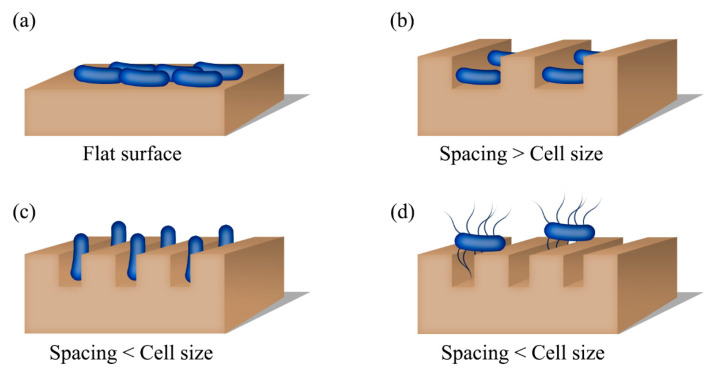
Microorganisms orient in different ways to overcome barriers that restrict the attachment to a surface. (**a**) The entire substrate is available for microbial attachment, (**b**) When the spacing or gap between the neighboring features is larger than the size of a cells, the microbial cell can lie between the features, (**c**) Gaps smaller than the size of a microbe force cells to stay on top of the features, decreasing the accessible surface area, and (**d**) Microbes with appendages like flagella can fit into the trenches to access the surface between the features.

Although surface patterns reduce the attachment of microorganisms, the opposite can occur depending on the organism. Ref. [128] and the stiffness of the material [89]. The attachment of *E. coli* was increased when the PDMS surface was patterned with arrays of microscale hexagonal features (height 2.7 μm and diameter 3 μm) separated by nanoscale trenches (440 nm) [128]. Here, the trenches were too small for the cells to fit into, but the cells used flagella to adhere to the surface. Since flagella can access nanoscale trenches, cells experience a higher surface area on patterned substrates than on flat samples. Consequently, patterned surfaces showed an increased attachment compared to the unpatterned analogues [128]. As well, the aggregation of *P. aeruginosa* cells was greater on the epoxy substrates containing HAR nanoposts [89]. The high aggregation of cells on a patterned surface can be attributed to the high stiffness of epoxy. Although studies show that stiff surfaces promote microbial attachment, little is known about the mechanosensing of prokaryotic cells [72]. Mechanosensing is the biological ability of cells and organisms to detect and respond to mechanical forces, such as pressure, stretch, and stiffness, in their environment—a well-established and crucial cellular function in eukaryotic cells. A study by Liyun Wang et al. found that surfaces with higher elasticity exhibit greater changes in mechanical stress and strain in the bacterial envelope than lower-elasticity surfaces. Adhesion to higher-elastic surfaces increases cyclic-di-GMP levels, a second messenger used in signal transduction in many bacteria, thereby reducing motility and decreasing detachment [131]. Figure 4 depicts the different orientations of microbial cells that maximize interactions between the cell and the substrate.

Overall, feature size and spacing between features control the attachment of microbes to a surface. The feature size and the gap between structures affect the attachment and ordering of the microbes, as well as the morphology and size of the cells. The cells alter their shapes and adopt various orientations to maximize the interactions with the substrate when attaching to the textured surfaces. Also, the characteristics of attaching cells, such as the presence of flagella, play an important role in adhering to a surface [106,127,128,132].

Surfaces engineered with subcellular-level features can also affect the morphology of the microbial aggregates. The micro- and nanoscale trenches patterned on a gold surface affect the adhesion of *P. fluorescens* cells and aggregation [127]. The cells formed well-ordered aggregates on the surfaces containing random nanostructures. The features with defined dimensions present on the substrates inhibited the formation of ordered aggregates.

Other studies report the use of biomimetic surface-patterned substrates to control microbial adhesion. One such pattern mimics the surface features present on the shark’s skin. PDMS elastomer with a topography similar to shark skin (Shark AF^TM^) exhibited lower attachment and aggregation of *S. aureus* than the unpatterned surfaces [108,126]. *S. aureus* was chosen for the study due to the matching sizes of cells and the Shark AF^TM^ features (width and spacing between the adjacent structures = 2 μm and depth = 2 μm). Also, it is noteworthy that most studies correlate the attachment of microbes to surface topography; however, in some cases, no apparent effect was found [133].

In addition to reducing the accessible surface area, nanostructures introduce bactericidal properties [125,132,134,135]. For example, sharp nanotopographical features patterned on the surface of silicon wafers killed the *E. coli* and *S. aureus* cells attached to the silicon surface [132]. The bactericidal effect was enhanced by coating the textured surfaces with chitosan, a biopolymer that has antibacterial properties. The spherical, Gram-positive *S. aureus* cells were attached on top of the nanostructures and between the structures, whereas Gram-positive, rod-shaped *E. coli* cells could not settle in between the nanostructures. Nevertheless, both types of bacteria deformed to achieve the best orientation on the patterned surfaces. Similarly, the bactericidal effect and reduced adhesion of *E. coli* were observed when the surface of poly(methyl methacrylate) (PMMA) films was patterned with nanopillars [125]. The optimum spacing between neighboring pillars was found to be in the range 130–380 nm. The bactericidal effect of a nanoengineered surface can be attributed to the cell lysis resulting from the high level of stress caused by the sharp tips of nanostructures [125]. Upon settling down on the nanostructures, the microbial cells stretch and deform. During this process, the sharp nanotips can rupture the cell walls and membranes, ultimately killing the cells [132,135]. The fabrication of nanostructured surfaces that can kill microorganisms is inspired by examples found in nature, such as cicada wings [136].

Decreased attachment of microbial cells on the patterned surfaces can be a result of the superhydrophobicity of the surface [128]. Structures on patterned surfaces may trap air in between spaces, making penetration of water (or other liquids) into the gaps difficult. So, the liquid droplets stay on top of the structures. This state is known as the Cassie-Baxter state. In that case, only the feature tops are available for the microbes, consequently reducing the attachment [128]. In the Cassie-Baxter state, the adhesion between the liquid droplet and the solid surface is low; therefore, the droplet can move easily on the surface. The surface is known to be superhydrophobic [69]. Same as the liquid droplets, any other particle, such as dirt or microbial cells, adheres weakly to such surfaces. A liquid droplet that rolls on a superhydrophobic surface may encounter a particle and carry the particle with the droplet, creating a self-cleaning surface [69]. Although the Cassie-Baxter state allows the liquid droplet to interact only with the feature tops, over time, the liquid can replace the air trapped between the structures. The wetting state transitions to another state known as the Wenzel state, allowing the microbial cells to reach the surface. At initial time points where the Cassie–Baxter conditions exist, the patterned surfaces exhibit a low microbial attachment. As time passes, the wetting state converts to the Wenzel state, the cells can reach the surface between the structures, and the attachment increases [128].

Inspired by nature, surfaces containing hierarchical patterns have been examined as a strategy that prevents colonization of microorganisms [137,138,139]. As reported in one study, the attachment of *P. aeruginosa* was decreased on a titanium surface patterned with hierarchical structures that mimic the surface topography of the lotus (*Nelumbo nucifera*) leaf [139]. In contrast, increased attachment of *S. aureus* was observed on the same surface pattern, indicating that the surface features alone cannot determine attachment of microorganisms. The difference in attachment was attributed to the shapes of the bacteria (rod-shaped *P. aeruginosa* vs. spherical *S. aureus*).

**Figure 5:**
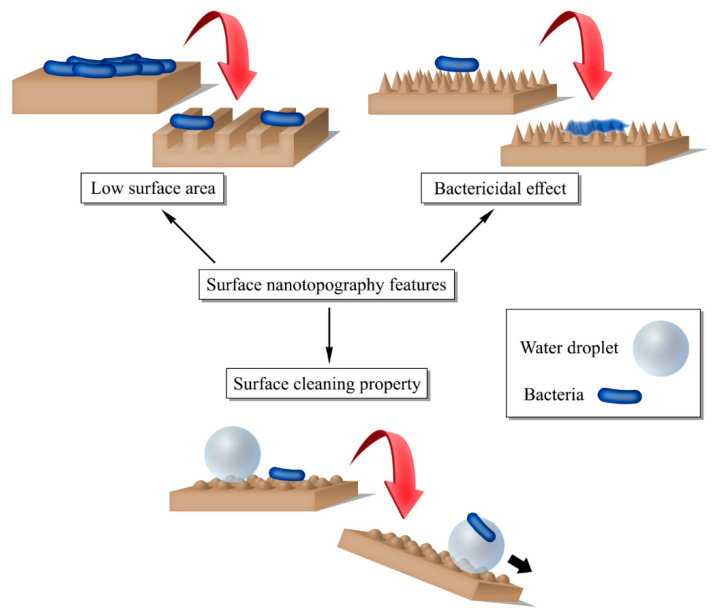
Different mechanisms through which nanopatterned surfaces combat biofilms.

In general, nanostructures on biomaterial surfaces control biofilms by reducing accessible surface area, promoting self-cleaning, or exerting bactericidal effects (Figure 5). Therefore, patterning surfaces with nanoscale topographies is an effective method for eliminating biofilms from biomaterial surfaces, without using antimicrobial agents or chemicals that can cause side effects.

## 5. Hydrogels as Biomaterials

Hydrogels are network polymers that can retain large quantities of water or biological fluids due to the network structure formed by crosslinking between the polymer chains [140]. By absorbing water, these polymer networks become gel-like soft materials that can mimic the natural biological environment. The properties of hydrogels can be easily tailored by adjusting parameters such as chemical composition, crosslinking, surface topography, and architecture to suit specific applications. Hydrogels have garnered significant interest as biomaterials due to their porous structure, tunable properties, soft nature, and favorable biological properties such as biocompatibility and biodegradability [141,142]. In particular, the biological applications of hydrogels are drug delivery systems, scaffolding materials in tissue engineering, wound dressings, prosthetic and wearable medical devices, and biosensors, to name a few [143,144,145,146]. Although some studies of hydrogels and biomaterials are still in the preliminary stages, various hydrogel-based products are commercially available [147]. Most of these applications require the biomaterial to stay in contact with the human body for extended periods. Hence, biofilm-related issues constitute a significant concern for hydrogel-based biomaterials. Nanotechnological strategies have been integrated to introduce antimicrobial properties into hydrogels. For instance, antimicrobial hydrogels have been fabricated by incorporating nanoparticles [148,149,150,151,152,153].

### 5.1. Current Status and Novel Trends in Surface-Patterned Hydrogels as Antibiofouling Materials

Hydrogels containing various topographical features have been extensively studied in biological and biomedical applications to control the attachment, functions, and behavior of cells [154,155,156,157,158,159]. Although nanopatterning has been explored as a method to mitigate biofilms on various materials, including silicon, titanium, and polymers, studies that involve nanopatterned hydrogels are limited. Since hydrogels are well-known for their biomaterial applications, it is essential to study nanopatterned hydrogels for antibiofouling applications. Being hydrophilic polymers, hydrogels possess antiadhesive properties as hydrophilic polymer coatings show low adhesion of microorganisms and biomolecules such as polymers [79]. Thus, patterning hydrogels with nanotopographic features will further enhance their intrinsic antiadhesive nature.

The surface patterns generated in most studies are static, meaning they remain unchanged over time. Maintaining the functionality of these static textures is challenging, as biological factors and interactions with cells may degrade the material’s original architecture, leading to loss of antibiofouling properties [20]. Currently, another technique known as dynamic topography, inspired by nature, is being investigated as a solution to overcome these challenges associated with the static topography approaches [160,161,162]. Dynamic topography is a bioinspired approach in which the surface and topographical features change repeatedly, subsequently facilitating delamination of bacteria attached to the surface [160]. Therefore, materials with dynamic topography are considered self-cleaning surfaces. The changes in the surface can be induced by external stimuli such as pH, electrical signals, temperature, etc. [162]. The ability to change the surface in response to external factors is an excellent property for an antibiofouling biomaterial. Hydrogels are popular stimuli-responsive materials, as these network polymers can change their swelling in response to external factors [163]. Moreover, the soft, gel-like nature of hydrogels is beneficial for controlling the formation of biofilms. As previously shown, the aggregation of bacterial cells to form biofilms can be inhibited by using arrays of nanoposts on soft polymers [89]. Considering the suitability of hydrogels for applications as biomaterials that are in contact with the human body, their soft nature, and their stimuli responsiveness, further research is necessary to develop hydrogels with dynamic nanotopographical features.

### 5.2. Fabrication of Surface-Patterned Hydrogels

**Figure 6:**
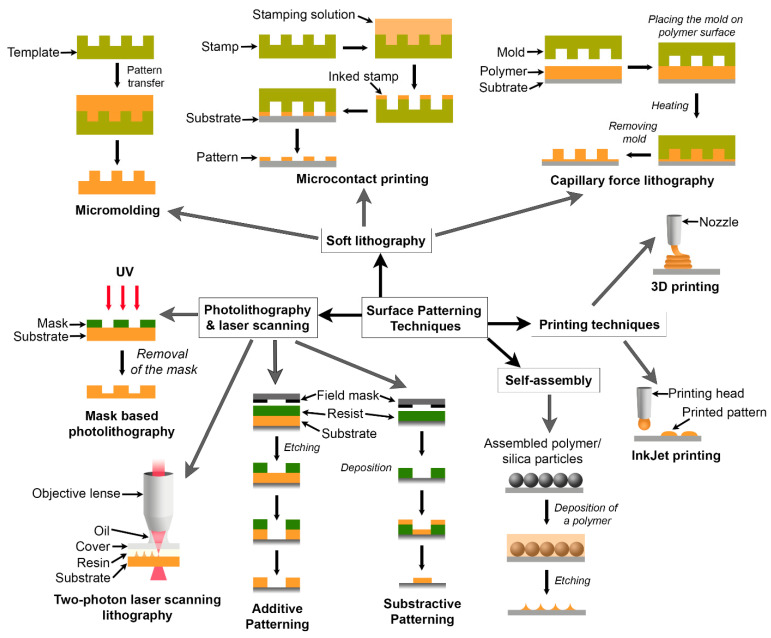
Widely used surface patterning techniques.

Fabrication of surface structures involves several techniques. Figure 6 shows some of the widely used techniques. In photolithography, a desired pattern is initially created on a mask (e.g., a silicon wafer), transferred onto a reactive polymer layer known as a resist, and subsequently replicated onto another substrate [164].

Photolithography is a standard method that is used to generate micron-level structures; however, fabrication of nanoscale features requires advanced lithography techniques such as deep ultraviolet (UV) and extreme UV photolithography, X-ray lithography, focused ion beam (FIB) lithography, and electron beam writing [165]. Since these advanced methods are expensive and require sophisticated conditions, a low-cost alternative known as soft lithography has been developed [165,166]. In soft lithography, the original pattern generated on a master material, such as a silicon wafer, is transferred to another substrate using an intermediate soft organic or polymeric material as the mold. Although there are various soft lithography methods, such as microcontact printing, embossing, replica molding, micromolding in capillaries (MIMIC), and microtransfer molding, not all of them can pattern nanoscale features. Among these different methods, replica molding and microtransfer molding can successfully pattern the delicate structures in the nanometer range [165]. In replica molding, the use of an elastomeric mold allowed the transfer of the pattern from the master, enabling the fabrication of complex structures with different sizes, shapes, and periodicities. Unlike the replica molding technique, which can pattern thick substrates, microtransfer molding can pattern only a thin film (thickness of 100 nm or less) of a polymer. Additionally, there are limitations on the materials that can be patterned using these lithography methods. For example, photolithography can directly pattern only polymers with photosensitive additives [166]. Various techniques for patterning polymers with micron and submicron-level topographical features have been reviewed [167]. Table 5 compares alternative lithography methods used for patterning hydrogels.

Patterning hydrogel surfaces is challenging due to their swelling properties, adhesion, and low mechanical strength [173,174]. Replica molding has been successfully used to pattern poly(2-hydroxyethyl methacrylate) (poly(HEMA))-based hydrogels with surface structures in the micrometer scale [175,176]. Swelling-induced delamination of the hydrogel from the PDMS mold ensures complete and uniform transfer of the pattern onto the hydrogel [175]. However, patterning hydrogels with nanofeatures may require adjustments to the existing methodologies, as the pattern transfer becomes more difficult with decreasing feature size. Therefore, the conditions should be optimized based on the hydrogel system and dimensions of the pattern. Recently published papers discuss the effects of surface patterns on properties and applications of hydrogels, and various methods and techniques used to create surface patterns [177,178]. Table 6 summarizes surface-patterned hydrogel systems.

### 5.3. Limitations of Surface-Patterned Hydrogels

Although hydrogels can be developed as antifouling biomaterials, their inherent properties can limit their applications. Hydrogels become soft when swollen in water. High water content and soft nature result in low mechanical properties, which may be incompatible with certain applications, such as orthopedic and dental implants [183]. As discussed in Section 5.2, producing hydrogels with nanoscale surface patterns is challenging because these fragile features can collapse. For example, nanostructures with high aspect ratios (HARs) may be unstable and collapse. Additionally, achieving high fidelity in the patterning process is another challenge [184].

## 6. Future Perspective

Patterning surfaces with nanotopographical features is a strategy that can prevent the formation of microbial biofilms on implants and indwelling medical devices. Nanopatterned materials inhibit the attachment of microorganisms and the aggregation of cells to form biofilms by altering surface properties, such as hydrophobicity and stiffness, and limiting available contact sites for cell interaction. The application of nanometer-scale surface features to alleviate attachment of microorganisms has been investigated across a broad range of materials, including silicon, metals and metal oxides, ceramics, and polymers. Nonetheless, the application of nanopatterning to develop antibiofouling hydrogels has not been widely studied, despite surface-patterned hydrogels being well known for their applications as biomaterials. Therefore, further research is needed to develop antifouling nanopatterned hydrogels. Patterning hydrogels with different nanoscale features is challenging yet worthwhile to study, as nanopatterned hydrogels offer a promising solution to biofilm-related problems associated with medical implants. Researchers should also consider the features of the target microorganism, such as type, size, and shape, when fabricating nanopatterned hydrogels for antifouling biomaterial applications. Furthermore, dynamic topography can be applied to hydrogels to restrict the attachment of microbes, simultaneously offering self-cleaning capability. Additionally, understanding the interactions of microorganisms with these soft polymeric materials is necessary to develop biomaterials that meet expectations, and is another important area to focus on.

A more modern approach to addressing problems associated with traditional surface patterning methods is to use machine learning. Machine learning can be used to optimize fabrication parameters, thereby improving reproducibility and scalability [185].

Overall, future research should focus more on optimizing existing techniques or developing new methods to fabricate stable, cost-effective, eco-friendly nanostructured hydrogels. Moreover, depending on the target application, hydrogel properties, such as mechanical strength, durability, and biocompatibility, must be optimized. The antifouling properties of nanostructured hydrogels can be enhanced by incorporating other materials, such as metal or polymer nanoparticles. There is a research gap in this area that needs more attention, as it has the potential to develop antimicrobial surfaces with structural features rather than relying on harmful chemicals.

## Figures and Tables

**Table 1:** Types of microorganisms that form biofilms on indwelling medical devices and implants.

Medical Device/Implant	Type of Microorganism	References
Urinary catheters	*E. coli*, *Providencia stuartii*, *Enterococcus*, *Pseudomonas*, *Enterobacter*, *Candida*, and *Serratia* species	[26,27,28]
Central venous catheters	*S. aureus*, *S. epidermidis*, *P. aeruginosa*, *Bacilli*, *Klebsiella*, and *Candida* species	[29,30]
Contact lenses	*P. aeruginosa*, *Serratia marcescens*, *S. aureus*	[31]
Prosthetic joints	*S. aureus*, *S. epidermidis*, *P. aeruginosa*, *E. coli*, *Streptococcus*, and *Enterococcus* species	[32,33]
Mechanical heart valves	*S. aureus*, *S. epidermidis*, *Streptococcus*, *Bacillus*, *Enterococcus*, and *Candida* species	[29]
Intravascular devices (cardiac pacemakers)	*S. aureus*, *S. epidermidis*, *Propionibacterium acnes*	[28,34]
Voice prostheses	*Rothia dentocariosa*, *Staphylococcus*, *Streptococcus*, *Escherichia*, *Enterobacter*, *Proteus*, *Pseudomonas*, and *Candida* species	[35,36,37]
Endotracheal tube	*S. aureus*, *S. epidermidis*, *Kocuria varians*, *Acinetobacter baumannii*, *P. aeruginosa*	[38,39]
Breast implants	*S. aureus* and *S. epidermidis*	[40,41]

**Table 2:** A comparison of different biofilms.

Bacterial Biofilms		Fungal Biofilms	Mixed (Polymicrobial) Biofilms
Gram-Positive	Gram-Negative		
Contains species such as *S. aureus*, *S. epidermidis*, *E. faecalis*, and *Streptococcus pneumoniae* [45]	Contains species such as *E. coli*, *K. pneumoniae*, *A. baumannii*, *P. mirabilis*, and *P. aeruginosa* [46]	Contains *Candida* species, including *C. parapsilosis*, *C. tropicalis*, *C. krusei*, and *C. glabrata*, and other species such as *Cryptococcus neoformans*, *Coccidioides immitis*, *Aspergillus* species, *Fusarium* species, *Blastoschizomyces capitatus*, *Malassezia pachydermatis*, *Pneumocystis* species, *Trichosporon asahii*, *Rhizopus* species, and *Rhizomucor* species [47]	Contain bacteria (Gram-positive/negative) and fungi, may contain viruses or algae [48,49]
Secrete polysaccharides, proteins, teichoic and lipoteichoic acids, membrane vesicles (MV), and extracellular DNA (eDNA) [50,51]	Secrete polysaccharides, amyloid proteins, lipopolysaccharides (LPS), outer membrane vesicles (OMVs), and specific types of amyloid fibers [51]	Polysaccharides, proteins, lipids, and Edna [52]. For example, biofilms of *C. albicans* contain proteins, chitins, DNA, and β-1,3 glucan carbohydrates. *C. neoformans* biofilms consist of xylose, mannose, glucose, and several minor sugars. *A. fumigatus* produces galactosaminogalactan and galactomannan as the major polysaccharides [53]	Compared to other types, they have higher biofilm masses, higher community cell count, enhanced metabolic activity, prominent antimicrobial tolerance, and changes in spatial organization and structure [54]
Can grow on catheters and prosthetic implants [45]	Can be found in water bodies and mammalian intestine [51]	Can grow on central urinary catheters, venous catheters, prosthetic valves, left ventricular assist devices, and oral devices, such as dentures [55]	Found in the oral cavity, mammalian intestines, and implantable medical devices [48]

Note: Gram-negative bacteria have lipopolysaccharides (LPS) in their cell membrane. In contrast, Gram-positive bacteria have teichoic acid (TA) in their cell wall. Both LPS and TA, which provide charges on the outer surface of bacteria, may influence bacterial adherence to surfaces. Therefore, these compounds may influence biofilm formation [51]. Both Gram-positive and Gram-negative bacterial biofilms are found in similar environments, such as moist surfaces with access to nutrients.

**Table 3:** Advantages and disadvantages of general methods used to prevent biofilms.

Method	Advantages	Disadvantages
Surface topographical modifications	Do not use antibiotics or any other drugs that can be toxic to host tissues (morphologically disturb microbial attachment). No antibiotic resistance. Do not affect the properties of the bulk material (e.g., biocompatibility)	There is a chance to form the biofilm if host proteins (in blood and serum) are attached to the surface.
Antimicrobial coatings (antibiotics, antimicrobial peptides (AMPs), etc.)	Active only on the site of application. Increases the concentration of the antimicrobial agent locally. Low chance of antimicrobial resistance when AMPs are used	Effective only for a short time due to the limited supply of the antimicrobial agent
Incorporation of antibiotics	Can locally increase the antibiotic concentration	May be effective only for a short period due to the depletion of materials. May cause local toxicity to the host tissues
Incorporation of nanoparticles	Preferential and controlled delivery over a long period of time. Delivery of multiple agents simultaneously improved efficacy	Can be incompatible and toxic to the host tissue. Environmental safety issues
Use of biological inhibitors	Some agents exhibit multiple effects, such as interfering with signaling pathways and destroying mature biofilms. Low toxicity to the host tissue	May become cytotoxic at high concentrations. Poor solubility and rapid metabolic degradation may cause issues.

**Table 4:** Mechanisms of biofilm inhibition by nanoparticles.

Type of Nanoparticles	Mechanism	Tested Organism	Refs.
Metal nanoparticles (e.g., Ag, Au, Pt, Ni, Cu, etc.)	Generation of ROS, disruption of biofilm matrix, membrane disruption, and inhibition of biofilm adhesion proteins	Various Gram-positive and negative bacteria, *S. aureus*, *S. epidermidis*, *E. coli*, *Bacillus subtilis*	[93,94,95,96,97,98]
Metal oxide nanoparticles (e.g., ZnO, Fe_3_O_4_, MgO)	Generation of ROS, Immune modulation, Disruption of established biofilms, Membrane disruption	*P. aeruginosa*, *S. aureus*, *E. coli*	[99,100,101,102]
Polymers (e.g., Chitosan-alginate)	Membrane disruption	*S. aureus*, *P. acnes*	[103]
Liposome-based nanoparticles	Target delivery of antibiofilm agents, Enhanced penetration	*S. aureus*	[104]
Composites (e.g., Berberine, silver nanoparticles, and carboxylated chitosan)	Membrane disruption	*S. aureus*	[105]

**Table 5:** A comparison of alternative lithography methods.

Patterning Technique	Advantages	Disadvantages	Refs.
Casting Crosslinking of the hydrogel in a patterned mold	Simple, economical, and versatile method. Has the potential to pattern non-planar surfaces	Limited to micron-sized patterns due to the low wetting properties between the hydrogel and the mold	[168]
Nanoimprint lithography (NIL) A hot embossing process conducted at high pressure	A low-cost, high-throughput patterning method	Limited use with hydrogel materials due to required high pressure and high temperature conditions, a complex imprinting process, high mold-fabrication costs, demolding that can cause pattern distortion or sticking, and molds that are prone to wear or contamination over repeated cycles.	[169]
Digital plasmonic patterning (DPP) Uses nanoparticles with plasmonic resonance to pattern a computer-generated pattern on hydrogel	Stiffness can be modulated by varying the laser intensity, writing speed, and digital pattern, thereby providing greater flexibility and eliminating the need for multiple polymer solutions or physical masks.	High cost Time consuming	[170]
Directed plasma nanosynthesis (DPNS) Uses Ion bombardment to generate nanostructures on hydrogels	Can fabricate high-precision nanostructures, a cost-effective, eco-friendly solution due to the use of non-toxic Argon plasma	Expensive	[171,172]

**Table 6:** A summary of surface-patterned hydrogel systems.

Polymer	Feature Size	Fabrication Method	Tested Organism	Ref.
poly(ethylene terephthalate) shark skin patterns combined with titanium dioxide (TiO_2_) nanoparticles	Mold 1: height: 3 µm, width: 2 µm, pitch: 4 µm Mold 2: height: 1.6 μm, width: 1.3 μm, pitch: 4 μm	Shark skin patterns were made with solvent-assisted soft nanoimprint lithography	*E. coli* K12 MG1655	[179]
Bacterial cellulose	493 nm (etched thickness)	Directed plasma nanosynthesis (DPNS)	None (Mentioned the potential of antifouling applications)	[180]
polyethylene glycol-based polymer grafted with 2-methacryloyloxyethyl phosphorylcholine (nano needles)	50 nm in tip diameter, 200 nm in bottom diameter, 300 nm in height, and 500 nm in center-to-center pitch	Ultraviolet (UV) replica molding technique	*E. coli*, *B. subtilis*	[181]
Chitosan hydrogel films with nanopillars	Pattern 1: Periodicity 320 nm, diameter 120 nm, height 230 nm, aspect ratio 1:9; pattern 2: Periodicity 500 nm, diameter 190 nm, height 400 nm, aspect ratio 2:1	solvent-assisted dropcast lithography	*P. aeruginosa* (bacteria) and *Fusarium oxysporum* (Fungi)	[182]

## Data Availability

This is a review paper and no new data was acquired.
